# Subtemporal approach for the resection of a midbrain cavernous malformation: evaluation of safe surgical corridors

**DOI:** 10.3171/2019.7.FocusVid.19135

**Published:** 2019-07-01

**Authors:** Thalia Estefania Sanchez Correa, David Gallardo Ceja, Diego Mendez-Rosito

**Affiliations:** Department of Neurological Surgery, Centro Médico Nacional 20 de Noviembre, ISSSTE, Mexico City, Mexico

**Keywords:** midbrain cavernous malformation, midbrain safe entry zone, subtemporal approach, video

## Abstract

Brainstem cavernous malformation management is complex due to its critical location and deleterious effect when bleeding. Therefore, every case should be thoroughly analyzed preoperatively. We present the case of a female patient with a midbrain cavernous malformation. A comprehensive anatomical and clinical analysis of the surgical corridors is done to decide the safest route. A subtemporal approach was done and the lateral mesencephalic sulcus and vein were important anatomical landmarks to guide the safe entry zone. Nuances of technique and surgical pearls related to the safe entry zones of the midbrain are discussed and illustrated in this operative video.

The video can be found here: https://youtu.be/vYA-IgiT2lU.

## Transcript

My name is Diego Mendez-Rosito from Centro Médico Nacional 20 de Noviembre in Mexico City and we are presenting the video titled “Subtemporal Approach for the Resection of a Midbrain Cavernous Malformation: Evaluation of Safe Surgical Corridors.”

We are presenting the case of a 52-year-old female patient without any previous history of illness. She presented to the ER with a history of severe headache of acute onset that caused neurological deterioration requiring intubation.

An immediate CT scan was done showing a midbrain hemorrhage with severe intraventricular extension, invading the lateral third and fourth ventricles as it is shown in the red arrows. This is causing acute hydrocephalus as it is shown by the expanded temporal horns and third ventricle as it is shown here. Due to this acute hydrocephalus, the patient has suffered acute deterioration. A ventriculostomy was placed. The patient required management in the ICU where periodical neurological examinations are mandatory to identify the onset of any pathological reflexes.

Then, an MRI revealed an acute hemorrhage in the midbrain, still with ventricular extension, causing perilesional edema. It is important to note that there is no exophytic or bulging of the hematoma to the pial surface of the brainstem, which would allow a color distinction in its pial surface (Xie et al., 2018).

A cerebral angiogram was done which was negative. The diagnosis is a midbrain cavernous malformation.

After 6 weeks, the patient is neurologically and metabolically stable. At this point, the neurological examination revealed a conscious and reactive patient without motor or sensitive deficit, but with horizontal diplopia and nystagmus.

Now that our patient is in good conditions, we can analyze the surgical approach since the hematoma is still in the subacute phase (Nathal et al., 2018).

Now, if we analyze the axial cut of the MRI, where the cavernous malformation is sitting, and we remember the neural tracts and nuclei of the mesencephalon (Giliberto et al., 2010). We know that the lesion is displacing critical tegmental structures.

Therefore, we must consider the safe entry zones to the midbrain. At this level, the three possible corridors are: coming from the anterior zone, through the anterior mesencephalic zone; coming from the lateral zone, through the lateral mesencephalic sulcus; or through the posterior zone, through the inter- or supra-/infracollicular region.

If we decided to access through the anterior zone, we would require a COZ transsylvian approach to reach the anterior mesencephalic zone (Abla et al., 2010). Due to the location of our cavernous malformation, this approach would put in risk the frontopontine, corticobulbar, and corticospinal tracts as well as the red nucleus, substantia nigra, and oculomotor nerve, which would cause hemiplegia, tremor, diplopia, and ptosis, being an unacceptable postoperative deficit.

If we decided to access through the lateral zone, we would require either a subtemporal approach or a lateral supracerebellar infratentorial approach according to the extension of the hematoma. Accessing through the lateral mesencephalic sulcus could cause contralateral vibratory and touch pressure deficit due to the injury of the medial lemniscus and the spinothalamic tracts.

Now, if we accessed through the posterior zone, we would require a supracerebellar infratentorial approach to reach the cuadrigeminal cistern and decide either to enter through the inter-/supra- or infracollicular corridors. Other than the venous limitations, accessing through these corridors could result in eye movement or auditory postoperative deficit.

Considering that our patient presented with horizontal diplopia and nystagmus, we did not want to put in more risk the eye movements so we decided to access the lateral zone through the subtemporal approach.

A preoperative lumbar drainage was placed and the patient was positioned in a Mayfield in the lateral position. A temporal craniotomy was done. The dura is opened carefully to avoid any lesions of the temporal lobe or its vascular drainage. At this point, extra removal of CSF from the lumbar drainage will help us access the subtemporal corridor with less retraction. It is crucial to understand the anatomy of the complex formed by Labbé and remove the arachnoidal attachments which could cause a tear during retraction.

A patty is placed to protect the temporal lobe and careful dissection is done following the tentorium until we find the free edge as it is shown here. At this point, it’s important to open the arachnoid membrane formed by ambient cistern and identify the anatomy and taking care of the trochlear nerve and important vascular structures.

It’s always crucial to have in mind the anatomical landmarks as it shown in this anatomic dissection that resembles the exact view of our surgical approach showing the cerebral peduncle, the lateral mesencephalic vein, and the lateral mesencephalic sulcus (Rhoton and Yagmurlu).

Now, going back to the surgery we can also identify the lateral mesencephalic vein and the lateral mesencephalic sulcus. These landmarks guide our planned safe surgical entry point. At this point, we do a small, gentle cortical opening and brainstem fibers are stretched until hemosiderin is visible as it is shown here. Once we have enough space, we remove the liquefied hematoma and the cavernous malformation in it in a piecemeal fashion. After part of it has been removed, it’s important to use the suction-dissection technique to free the residual as it is shown here, which will allow an easier removal of the cavernous malformation. After we completed the removal it’s important to observe the surgical bed to try to identify any residual in any corner because this will be the cause of rebleeding. After we are positive that there is no residual we place a hemostatic agent. Care is taken in the temporal lobe and vein of Labbé before the closure.

A postoperative MRI revealed a gross-total removal of the cavernous malformation. The patient was discharged in the third postoperative day without any further complications. In the follow-up the patient resolved the preoperative visual disturbances and presented with only contralateral sensitive alterations which allowed her to fully recover.

The authors have permission to reproduce figures from the following sources:

02:41 Giliberto G, Lanzino DJ, Diehn FE, Factor D, Flemming KD, Lanzino G. Brainstem cavernous malformations: anatomical, clinical, and surgical considerations. **Neurosurg Focus 29 (3):**E9, 2010. © 2010 AANS.

06:47 Rhoton Collection (http://rhoton.ineurodb.org), CC BY-NC-SA 4.0 (http://creativecommons.org/licenses/by-nc-sa/4.0).

### Time points

0:42 Clinical presentation

0:57 Preop CT scan

1:38 Prep MRI

2:34 Discussion

5:22 Positioning

5:33 Surgery

6:47 Anatomical correlation

8:59 Postop MRI

9:13 Follow-up
